# The Impact of Education on Patients Eligible for Cardiac Rehabilitation and Factors Contributing to Declining Participation in Turkish Society: Are Patients Aware of Cardiac Rehabilitation?

**DOI:** 10.7759/cureus.62508

**Published:** 2024-06-17

**Authors:** Akin Torun, Berrin Topcu, Busra Z Buyukkilic, Sahhan Kilic, Irem Yilmaz, Mehmet Uzun

**Affiliations:** 1 Department of Cardiology, Sultan II. Abdulhamid Han Training and Research Hospital, Istanbul, TUR; 2 Department of Sports Medicine, Sultan II. Abdulhamid Han Training and Research Hospital, Istanbul, TUR; 3 Hamidiye Faculty of Nursing, Health Science University, Istanbul, TUR

**Keywords:** chronic heart failure, chronic coronary syndromes, patient education, cardiac rehabilitation adherence and participation, cardiac rehabilitation

## Abstract

Objective: Despite their effectiveness, cardiac rehabilitation (CR) programs have low participation and adherence rates. CR participation and adherence are directly related to the social, economic, cultural, and geographical characteristics of the society. Therefore, our study aimed to investigate the reasons behind low participation in CR within Turkish society, as well as the barriers that restrict participation despite educational efforts.

Method: The research was conducted with participants who were over 18 years of age, had any history of heart disease, and had been hospitalized in the last year. The patients' medical history, chronic diseases, demographics, habits, employment and income status, educational status, and approaches to CR were surveyed. Additionally, patients who still did not consider participating in CR after receiving information were asked about the reasons for their decisions.

Results: Although 95.6% of patients were eligible for CR, 91.9% of them were previously unaware of this treatment option. After being informed, 29.4% of patients agreed to participate in CR. The most common reasons for not participating after receiving information were as follows: three days a week is too much (21.9%); this place is far away, but if it were closer, I would come (18.1%); I can't come on weekdays (15.6%); and I would come if someone brought me (14.4%).

Conclusion: We observed that the participation rate in CR increased from 0% to 29.4% after receiving information. Furthermore, it was determined that the CR schedule and transportation were significant factors influencing participation.

## Introduction

Participating in cardiac rehabilitation (CR) considerably reduces morbidity and mortality rates and improves quality of life after various cardiac diagnoses and treatments. Cardiac rehabilitation aims to enhance strength, improve exercise endurance, facilitate return to work or daily activities, reduce symptoms like chest pain and shortness of breath, optimize cardiovascular risk factors, and prevent deterioration of heart disease and life-threatening incidents by increasing life expectancy [1]. CR programs are carried out by a multidisciplinary team with methodologies consisting of aerobic and resistance exercise combinations. Despite its effectiveness, cardiac rehabilitation programs have low participation and adherence rates [2]. 

In a large-scale study, patients eligible for CR were examined, and CR participation was found to be 24.4% [3]. Factors such as male gender, white ethnicity, older age, higher socioeconomic status, having insurance, receiving a strong recommendation from a physician, higher education levels, spousal support, lower cost-sharing, and closer proximity to the program have all been linked to higher rates of participation in cardiac rehabilitation [4]. Non-participants in CR were more inclined to be female, black, and had lower levels of education [5]. However, almost all of the studies on CR participation and adherence in the literature are based abroad. CR participation and adherence are directly related to the social, economic, cultural, and geographical characteristics of the society. Therefore, in our study, we investigated the reasons for the low participation in CR programs in Turkish society.

## Materials and methods

The research was conducted between March 2022 and December 2023 with participants over the age of 18 who had any history of heart disease. The study was conducted in patients currently hospitalized for cardiological disease. Additionally, every patient had at least one history of hospitalization within the previous year. Participants who met these inclusion criteria were consecutively included in the study. Hospitalizations that included single-day treatment and follow-up for various reasons were not included in the study. The study was conducted in a single center at a state hospital. The researchers obtained approval from the local ethical research committee. The study was conducted in accordance with the ethical principles outlined in the Helsinki Declaration, and informed consent was obtained from the patients. Additionally, artificial intelligence (AI)-supported technologies were not used in the production of the presented work.

An optimal environment was prepared for the patients, and they answered the survey questions themselves without any guidance. All patients were cooperative and oriented. Simple and understandable expressions were used in the questions. The patients' medical history, chronic diseases, demographics, habits, employment and income status, educational status, and approaches to CR were questioned. Furthermore, after the survey, patients were informed about CR and their opinions were requested regarding their potential participation in the CR program again. The purpose of CR, its possible benefits to the patient, its potential risks, the exercise and life habit interventions included in the program, and the program schedule were explained to the patients during the CR education interview. The information session lasted at least 15 minutes, and all questions from the patients were answered. After the education session, patients who still did not consider participating in CR programs were asked about the reasons for their decisions. Patients were able to choose one or more reasons for this issue.

The data analysis was conducted using IBM SPSS Statistics for Windows, Version 27 (Released 2023; IBM Corp., Armonk, New York). The Kolmogorov-Smirnov test was used to assess the normality of the data. The categorical data were presented using numbers and percentages. To compare categorical variables between groups, Fisher’s exact test or the two-tailed test was used, depending on the condition. The variables were displayed in the median (minimum-maximum) configuration. The categorical variables were represented numerically and as percentages, derived from the total count of participants for whom data were accessible. Statistical significance was defined as a p-value less than 0.05.

## Results

A total of 160 patients, 98 males and 62 females, were included in the study. The marriage status, residents, employment and income status, education, internet and social media usage, and social support status of the patients are presented in Table 1.

**Table 1:** Life characteristics of the participants

Characteristics	Value
Marital status	
Married	135 (84.4%)
Single	15 (9.4%)
Divorced/widow	10 (6.3%)
Cohabitants	
Wife/husband	132 (82.5%)
Alone	7 (4.4%)
With children	13 (8.1%)
With caregiver	2 (1.3%)
Nursing home	6 (3.8%)
Employment status	
Yes	128 (80%)
Retired	21 (13.1%)
Housewife	11 (6.9%)
Unemployed	0 (0%)
Income status	
Income exceeds expenses	42 (26.3%)
Income equal to expenses	95 (59.4%)
Income less than expenses	22 (13.8%)
Education	
University or more	15 (9.4%)
High school	40 (25%)
Middle school	30 (18.8%)
Primary school	64 (40%)
Literacy, no diploma	8 (5%)
No literacy	3 (1.9%)
Internet	
Yes	45 (27.5%)
No	115 (71.9%)
Social media	
No	49 (30.6%)
Yes	111 (69.4%)
WhatsApp	100 (62.5%)
Facebook	77 (48.1%)
Twitter (X)	10 (6.3%)
YouTube	48 (30%)
Social support	
Yes, whenever I want	26 (16.3%)
Yes, mostly	47 (29.4%)
Sometimes	15 (9.4%)
Rarely	46 (28.7%)
Never	25 (15.6%)

Male and female patients are compared in Table 2. Demographics, heart and other disease history, and risk factors were examined. Statistical differences were found only in body mass index and smoking between the two genders (p-values < 0.001 and 0.003, respectively).

**Table 2:** Participants' life habits and medical histories

Variables	Female (62)	Male (98)	P value
Age, median (min-max)	64.5 (51-82)	64 (37-87)	0.652
Height, median (min-max)	160 (147-175)	172 (150-188)	<0.001
Weight, median (min-max)	75 (50-101)	80 (59-110)	0.012
Body mass index, median (min-max)	29 (21-40)	26.5 (19-38)	<0.001
Cardiovascular diagnoses			
Coronary artery disease	47 (75.8%)	84 (85.7%)	0.113
Heart failure	12 (19.4%)	17 (17.3%)	0.748
Cardiomyopathy	1 (1.6%)	2 (2%)	0.846
Coronary bypass surgery	4 (6.5%)	10(10.2%)	0.413
Heart valve disease	4 (6.5%)	2 (2%)	0.153
Congenital heart disease	0 (0%)	2 (2%)	0.258
Arrhythmia	2 (3.2%)	10(10.2%)	0.103
Comorbid diseases			
Cancer	2 (3.2%)	0 (0%)	0.074
Arthritis	3 (4.8%)	2 (2%)	0.322
Diabetes mellitus	28 (45.2%)	34 (34.7%)	0.185
Cerebrovascular disease	1 (1.6%)	2 (2%)	0.846
Chronic obstructive pulmonary disease	4 (6.5%)	11 (11.2%)	0.313
Peripheral artery disease	1 (1.6%)	1 (1%)	0.742
Peripheral venous disease	8 (12.9%)	5 (5.1%)	0.078
Risk Factor			
Hyperlipidemia	32 (51.6%)	36 (36.7%)	0.064
Hypertension	42 (67.7%)	53 (54.1%)	0.087
Mental stress	15 (24.2%)	16 (16.3%)	0.220
Sedentary life	16 (25.8%)	21 (21.4%)	0.552
Smoking	13 (21%)	43 (43.9%)	0.003
Alcohol	2 (3.2%)	11 (11.2%)	0.071
Anxiety	10 (16.1%)	9 (9.2%)	0.186
Family history of heart disease	19 (30.6%)	18 (18.4%)	0.073

Table 3 presents patients' eligibility for the CR program, indications, information about CR, and their approaches. Although 95.6% of patients met the criteria for cardiac rehabilitation (CR), 91.9% were previously unaware of this therapy option. After being informed, 29.4% of patients stated that they were considering participating in the CR program. The most common reasons for not participating after receiving information were as follows: 21.9% of the patients marked "three days a week is too much"; 18.1% of the patients marked "this place is far away, but if it were closer, I would come"; and 15.6% said "I can't come on weekdays, I would come on the weekend." Among the most common answers given by the patients, reasons related to transportation and the CR schedule were noteworthy.

**Table 3:** Participants' approaches to cardiac rehabilitation

Variables	Values
Is the patient eligible for rehabilitation?	
Yes	153 (95.6)
No	7 (4.4)
If the answer is yes, indication?	
Heart valve disease	8 (5%)
Coronary artery disease	116 (72.5%)
Heart failure	22 (13.8%)
Recent coronary artery bypass surgery	10 (6.3%)
If the answer is no, why?	
No cooperation	5 (3.1%)
Rehabilitation has been done before	0 (0%)
Not an indication	2 (1.3%)
Do you know cardiac rehabilitation?	
Yes	13 (8.1%)
No	147 (91.9%)
If you know about cardiac rehabilitation, where did you learn it?	
Nurse	1 (0.6%)
Doctor	3 (1.9%)
Physiotherapist	0 (0%)
Television	3 (1.9%)
Other patients	0 (0%)
Internet	6 (3.75%)
From my relatives who have participated before	0 (0%)
Other	0 (0%)
In your opinion, which of the following does Cardiac Rehabilitation include?	
No idea	91 (56.9%)
Exercise	35 (21.9%)
Information about heart	45 (28.1%)
Coronary angiography	13 (8.1%)
Stent or balloon placement in the heart arteries	4 (2.5%)
Teaching ways to prevent stress	16 (10%)
Performing echocardiography on patients	21 (13.1%)
Organizing the treatment of patients	23 (14.4%)
Education of patient relatives	1 (0.6%)
Nutrition education	22 (13.8%)
Teaching patients how to cook	1 (0.6%)
Heart screening of patient relatives	5 (3.1%)
Information about hypertension, diabetes, and cholesterol	15 (9.4%)
Information about the harms of smoking	14 (8.8%)
Psychological support	24 (15%)
Treatment of stroke patients only	0 (0%)
Heart health check of athletes	2 (1.35)
Frequent treadmill testing	9 (5.6%)
We think cardiac rehabilitation would be beneficial for you. Would you consider participating?	
Yes	47 (29.4%)
If your answer is no, why?	
I don't believe in its benefit	13 (8.1%)
I'm working, I can't come	17 (10.6%)
This place is far away, if it were closer I would come	29 (18.1%)
I can't come on my own, I would come if someone brought me	23 (14.4%)
It might help but I don't want it	19 (11.9%)
I have other illnesses, I need to take care of them first	15 (9.4%)
Three days a week is too much	35 (21.9%)
I can't come on weekdays, I would come on the weekend	25 (15.6%)
I'm taking care of a child, I can't come	12 (7.5%)
I don't want to do sports in the same place as men (For women)	6 (3.8%)
I don't want to do sports in the same place as women (for men)	0 (0%)
I already exercise regularly	0 (0%)
I don't have money to cover my travels	0 (0%)

## Discussion

CR includes a variety of interventions aimed at improving the quality of life for individuals diagnosed with cardiovascular diseases through a combination of education, physical activity, and behavioral modifications. Studies show CR programs have the potential to decrease both mortality and morbidity rates subsequent to a cardiac event while also enhancing quality of life and psychological well-being [6]. Despite all its benefits, participation rates in CR programs globally are not at the desired level. Annually, almost 88,000 individuals in the UK initiate CR [7]. A study conducted in the United States revealed that the overall participation rate in CR was 24.4% [3]. However, there is no clear data yet regarding the participation in CR programs in Türkiye. Therefore, our study may be useful in increasing CR participation and adherence.

For the first time in Türkiye, the population over the age of 65 will exceed 10% in 2023. According to population projections, the elderly population rate is expected to be 12.9% in 2030, 16.3% in 2040, 22.6% in 2060, and 25.6% in 2080 [8]. CR is an ideal treatment approach for elderly patients [9]. The aging society and the increasing prevalence of cardiovascular disease make rehabilitation programs a mandatory target. Evidence also suggests that CR is cost-effective, especially with exercise as a component. Moreover, foreign countries were evaluated in these studies, and the cost per patient is quite high compared to Türkiye.

One of the striking data points in the study results is that although the average age is 64, 80% of the participants are employed. Economic reasons can be considered a factor for this situation. Because 73.2% of the participants responded to the question about their income that their income was equal to or less than their expenses. Another interesting data point among heart disease patients is that while 71.9% say no to internet use, the rate of saying yes to social media use is 69.4%, regardless of the first question. These results show that most internet usage is actually related only to social media. Considering the widespread use of social media, it can be a tool for providing information about CR programs.

When we compared the clinical characteristics of men and women participating in the study, women had high body mass indexes and the smoking rate was high in men. Similarly, in a previous study on coronary artery disease in Türkiye, the smoking rate in women was low and their body mass index was high [10]. Our study was evaluated as compatible with national data in the literature.

The study found that 95.6% of the participants were eligible for CR. This rate is consistent with data from other countries [4]. It is noteworthy that 91.9% of these patients who had been hospitalized at least once in the last year did not have any information about CR. An ambitious goal of increasing CR participation from 20% to 70% has been set in the United States [11]. Although these rates do not seem possible in the short term for Türkiye, initial steps may involve informative approaches. We observed that the rate of those who said they would participate in the CR program increased from 0% to 29.4% after receiving information. In a study conducted in outpatient clinics in Türkiye, it was observed that one of the notable reasons for not participating in the CR program was that patients did not believe in the benefits of CR [12]. In our study, we found that this rate decreased to 8.1% with information.

The program's three-day schedule, which includes weekdays, has been recognized as a barrier to participation for a significant percentage of patients. A potential solution to this issue could be home-based CR. The efficacy of home-based CR programs has been a subject of debate in the past. However, recent research indicates that both home-based (including digital/telehealth platforms) and center-based forms of cardiac rehabilitation, when formally endorsed by healthcare professionals, demonstrate comparable effectiveness in enhancing clinical and health-related quality of life outcomes [13].

When we examine the reasons for not being able to participate, transportation problems are one of the most common answers. This issue can be addressed by increasing the number of health institutions with available CR centers. Another transportation-related reason is that patients wish to participate but have no one to assist them with transportation. The main factor for this situation may be that 53.7% of patients sometimes, rarely, or never have access to social support. The solution may be a social support service based on a CR program for chronic heart diseases.

In our study, we included patients with a history of hospitalization due to cardiac disease and investigated their attitudes about CR programs. Our research has shown that education can lead to a significant increase in CR participation among eligible patients. Additionally, reasons for not being able to participate are presented. This data may provide a new evidence base for policymakers.

Limitations

The study was conducted with patients living in Istanbul. Although Istanbul is a city that receives immigrants from every region of Turkey, it is not clear how much it represents the entire Turkish population. Future studies involving all geographical regions with more participants may provide detailed information about CR participation and adherence. The study did not account for potential confounding factors such as the severity of participants' heart conditions or comorbidities. Since it is an initial study, basic data in all patient groups is targeted for future detailed studies. Another limitation is that the data are based on self-report. In this regard, patients were provided with an optimal environment, and a simple and understandable language was used without any pressure or guidance.

## Conclusions

Education of patients about CR programs is not sufficient and should be the first approach. We observed that the participation rate in the CR program increased from 0% to 29.4% after the information. Additionally, schedule and transportation were observed as barriers to participation. These results show the necessity of increasing social support and the need for further studies on home-based CR.
